# A Multi-Information Spreading Model for One-Time Retweet Information in Complex Networks

**DOI:** 10.3390/e26020152

**Published:** 2024-02-09

**Authors:** Kaidi Zhao, Dingding Han, Yihong Bao, Jianghai Qian, Ruiqi Yang

**Affiliations:** 1 School of Information Science and Technology, Fudan University, Shanghai 200433, China; 21210720091@m.fudan.edu.cn; 2 Shanghai Artificial Intelligence Laboratory, Shanghai 200232, China; 3 School of Mathematics and Physics, Shanghai University of Electric Power, Shanghai 201306, China; qianjianghai@shiep.edu.cn; 4 School of Communication and Electronic Engineering, East China Normal University, Shanghai 200062, China; 51161214037@stu.ecnu.edu.cn

**Keywords:** multi-information spreading, online social network, inhibiting factor, enhancement factor, one-time retweet information

## Abstract

In the realm of online social networks, the spreading of information is influenced by a complex interplay of factors. To explore the dynamics of one-time retweet information spreading, we propose a Susceptible–Infected–Completed (SIC) multi-information spreading model. This model captures how multiple pieces of information interact in online social networks by introducing inhibiting and enhancement factors. The SIC model considers the completed state, where nodes cease to spread a particular piece of information after transmitting it. It also takes into account the impact of past and present information received from neighboring nodes, dynamically calculating the probability of nodes spreading each piece of information at any given moment. To analyze the dynamics of multiple information pieces in various scenarios, such as mutual enhancement, partial competition, complete competition, and coexistence of competition and enhancement, we conduct experiments on BA scale-free networks and the Twitter network. Our findings reveal that competing information decreases the likelihood of its spread while cooperating information amplifies the spreading of mutually beneficial content. Furthermore, the strength of the enhancement factor between different information pieces determines their spread when competition and cooperation coexist. These insights offer a fresh perspective for understanding the patterns of information propagation in multiple contexts.

## 1. Introduction

The emergence of online social networks has revolutionized the spreading of information. These platforms provide easy access to a wide range of information, including news, entertainment, and academic research. This accessibility not only enhances people’s understanding of the world, but also facilitates the sharing and spread of knowledge, contributing to societal development and progress.

However, the rapid spread of information through online networks also brings about various challenges. Privacy breaches, exposure to false information, and the propagation of harmful rumors are among the negative impacts associated with this phenomenon [1,2,3,4,5,6]. Therefore, it is crucial to deeply explore different forms of information spreading. Understanding the mechanisms of information diffusion and implementing effective measures to mitigate its negative effects are essential for public opinion monitoring and information recommendation systems.

Drawing parallels between the spread of epidemics and information, researchers have often employed epidemic models to study information spreading. Previous research primarily focused on single information spread within networks [7,8,9,10,11,12,13,14]. However, given the vast array of information types and complex interactions within online social networks, which significantly impact the dynamics of information diffusion, scholars have increasingly turned their attention to multi-information spreading models. These models aimed to simulate and comprehend the intricate mechanisms underlying information propagation in online social networks [15,16,17,18,19,20,21,22].

The commonly used epidemic models, such as SIR and SIS, have been applied to the research of single information spreading [23,24,25,26]. Zhu [27] introduced the concept of information priority and focused on the competitive relationship between original false information and updated information. Shang [28] investigated the influence of overlap among communities on epidemics. By constructing an SIS model, it was found that epidemics spread faster in networks with a higher level of overlapping communities. Wang [29] studied the information spreading dynamics on complex networks with cliques and proved that the phase transition was always continuous and independent of the cliques. Continuous phase transition implies that the total number of infections during an epidemic varies with the infectiousness, exhibiting more complex collective dynamics influenced by environmental factors, including discontinuous or explosive transitions [30]. Prakash [15] proposed an information competition spreading model and discovered that strong information with a high spreading probability completely supplanted weaker information. Beutel [31] investigated the conditions for the coexistence of two competing viruses (or products or ideas) in a network. Wei [32] examined the intertwined diffusion of two rival information in a composite network based on the SIS model. They established a non-linear dynamic system and conducted an eigenvalue analysis to identify the critical point of epidemic behavior. Fátima [17] used the SIS model to explore the interaction between the spread of epidemics and the spreading of disease knowledge information, showing that the knowledge of the disease helped to reduce the disease prevalence and increase the epidemic threshold of the disease. Zhang [18] proposed an information diffusion framework to analyze the overall interactive behavior of users. Xiao [19] explored the impact factor and the interrelation of network layers on the information diffusion process based on the SIS model. Fan [33] studied the impact of a hybrid information propagation model on the epidemic and its related information coupling dynamics in a multilayer network. In this multilayer network, the upper layer was composed of randomly generated simple complexes, while the lower layer consisted of either traditional scale-free or Erdős–Rényi (ER) random networks.

However, despite extensive research on multi-information spreading models, there are still several unresolved issues that warrant attention.

1. When examining the relationships between different types of information, it is important to acknowledge that cooperation and competition can coexist within a network. While many researchers tend to focus on either cooperation or competition, understanding the interplay between these two dynamics is crucial. Even when two pieces of information compete, the degree of competition can vary, ranging from complete to partial. By considering the dynamics of spreading between different types of information, we can enhance our theoretical foundation for managing and controlling information spreading in networks. This deeper understanding allows us to develop strategies and measures that effectively address the negative impacts associated with information spreading. By recognizing the intricate interactions and complex nature of information propagation, we can establish more robust frameworks for managing and mitigating the consequences of network information spread.
2. Certain types of information possess strong timeliness, users may choose to retweet a piece of information upon first encountering it, but then cease to engage in any further retweets of the same content. Integrating the SIR model with one-time retweet behavior is crucial for accurately simulating and understanding the mechanisms of information spreading in social networks. In terms of its impact on information spreading, this ‘one-time retweet’ behavior could inhibit further diffusion of information, affecting the speed and breadth of information propagation. Therefore, this user behavior should be considered when constructing models of information propagation.

To address the above issues, we draw on the concept of influence factors [19] and propose a Susceptible–Infected–Completed (SIC) information-spreading dynamic model. We use the microscopic Markov chain approach (MMCA) [34,35] to establish the dynamic equation and discuss the information spreading mechanism under different relationships in both artificial and real networks. The main research results of this paper are as follows:

1. Proposal of the SIC model: We introduce a novel SIC model where nodes transition to a completed state after participating in information spreading. This model specifically focuses on one-time retweet information.
2. Examination of the impact factor: We incorporate the concept of influence factors to describe the interactions between different types of information. By considering free spreading, cooperative spreading, partial competitive spreading, complete competitive spreading, and the coexistence of competition and cooperation, we analyze how multiple pieces of information propagate within the network.
3. Utilization of the MMCA: We employ the MMCA to establish the dynamic equation of the SIC model. This mathematical framework allows us to dissect and understand the intricate dynamics of information spreading within the network.

By leveraging influence factors and employing the MMCA approach, we present a comprehensive analysis of information diffusion, shedding light on its mechanisms under diverse relationship scenarios. The proposed model is applied to analyze the spread of information in both simulated artificial networks and real-world network data.

The rest of this paper is organized as follows. In Section 2, we provide the problem definition. In Section 3, we describe the proposed model in detail. In Section 4, we present and analyze the experimental results of the model. Finally, in Section 5 we conclude the paper.

## 2. Problem Definition

In order to study the spreading of multiple pieces of information in a network and their interactions with each other, we first define a network $G=\left\{V,E\right\}$. $V=\left\{v_{1},v_{2},v_{3},\ldots ,v_{N}\right\}$ represents the set of nodes in the network, $v_{i}$ is specific nodes, and $E=e_{ij}=\left\{\langle v_{i},v_{j}\rangle |i,j\in V\right\}$ represents the edges in the network, with $e_{ij}$ indicating the edges between node *i* and node *j*. Information can spread along these edges. $Inf=\left\{Inf_{1},Inf_{2},\ldots ,Inf_{M}\right\}$ is employed to denote the collection of *M* distinct pieces of information.

In this paper, the focus is on analyzing the spreading of information in a network that has a lasting impact once it is disseminated. To address this, the proposed model is called the SIC model. The node status is divided into three categories: *S* (Susceptible), *I* (Infected), and *C* (Completed). Nodes in the $S_{i}$ state refer to those who have not yet been exposed to $Inf_{i}$ and are susceptible to its influence. Nodes in the $I_{i}$ state indicate those who receive $Inf_{i}$ from their neighbors and are interested in it. They are willing to spread $Inf_{i}$ to their friends. Nodes in the $C_{i}$ state indicate that the node has already spread a certain $Inf_{i}$ and maintains a memory of it, but the $Inf_{i}$ will no longer be spread by them. This state is similar to the “recovered” state in the SIR model. However, nodes in the $C_{i}$ state only mean that the node will no longer spread $Inf_{i}$, but there is still a possibility of spreading other information on the network. Moreover, because nodes in the $C_{i}$ state maintain a memory of $Inf_{i}$, this affects the probability of the node spreading other information in the future.

Table 1 displays the symbols used in our paper. The cooperative and competitive relationships between information are controlled by parameters $a_{1}$ and $a_{2}$. $a_{1}$ represents the influence factor between information in a competitive relationship, with $0\leq a_{1}\leq 1$. The larger the value of $a_{1}$, the greater the inhibit strength. When $a_{1}=1$, it means that the information completely repels other information, and the node only accepts certain information that repels others. When $a_{1}=0$, the information does not compete with other information. $a_{2}$ represents the influence factor between information in a cooperative relationship, and the larger the value of $a_{2}$, the stronger the cooperative strength. We use $\beta =\left\{\beta_{1},\beta_{2},\ldots ,\beta_{M}\right\}$ to represent the infection rate, where $\beta_{i}$ denotes the infection rate of $Inf_{i}$. The infection rate is defined as the probability that a node in the susceptible state (S state) will be influenced by a neighboring node in the infected state (I state)

## 3. Proposed Model

In this paper, the focus is on discussing five different types of information spreading within networks. These categories include free spreading, mutual enhancement, partial competition, complete competition, and the coexistence of competition and enhancement.

(1) Free spreading: In this scenario, there is no mutual influence between different pieces of information. Each piece of information spreads independently throughout the network without any direct interaction or impact from other information items.
(2) Mutual enhancement: When information spreads through mutual enhancement, the probability of a node spreading cooperative information increases after it disseminates a piece of information. This means that the act of spreading one piece of information enhances the likelihood of that node also spreading additional cooperative information.
(3) Partial competition: In the case of local competition between information, the probability of a node spreading competitive information is inhibited after it spreads a piece of information. This implies that when multiple pieces of information compete, the spreading of one piece of information inhibits the node’s tendency to spread other competitive information.
(4) Complete competition: when there is complete competition between information, a node will not spread competitive information after it spreads a piece of information.
(5) Coexistence of competition and enhancement: in the case of the coexistence of competition and enhancement between different kinds of information, the probability of a node spreading cooperative information increases, and at the same time, the probability of spreading competitive information is inhibited after it spreads a piece of information.

By exploring these different types of information spreading and their dynamics, we aim to provide a comprehensive understanding of how information disseminates within networks. This knowledge can contribute to the development of effective strategies for managing and controlling the spread of network information.

The Markov chain approach is utilized in this paper to analyze the five types of information spreading mentioned earlier. To facilitate discussion and explanation, let us consider an example with three pieces of information. The principles can be extended to include more than three pieces of information.

In this model, the initial node can receive any of the M pieces of information present in the network. For the sake of simplicity, we refer to the three pieces of information as $Inf_{1}$, $Inf_{2}$, and $Inf_{3}$. For further analysis, the subsequent content assumes that $Inf_{1}$ and $Inf_{3}$ have a cooperative relationship, while $Inf_{2}$ has a competitive relationship with both $Inf_{1}$ and $Inf_{3}$. We will explore their relationships within the context of the SIC model. Here are some examples of states within this model: when a node is in the state of $S_{1}S_{2}S_{3}$, it indicates that the node has not received any of the three pieces of information; when a node is in the state of $I_{1}S_{2}S_{3}$, it means that the node has only spread $Inf_{1}$; when a node is in the state of $C_{1}I_{2}S_{3}$, it means that the node has already spread $Inf_{1}$, and now it spreads $Inf_{2}$, which has a competitive relationship with $Inf_{1}$; and when a node is in the state of $C_{1}S_{2}I_{3}$, it means that the node has already spread $Inf_{1}$, and now it spreads $Inf_{3}$, which has a cooperative relationship with $Inf_{1}$. These states and their relationships are part of the SIC model, which aims to analyze the spread of information in a network while considering the effects of cooperation and competition among different pieces of information.

When the infection rate of $Inf_{i}$ is $\beta_{i}$, if the node is in $S_{1}S_{2}S_{3}$ state at time *t*, it will change to $I_{1}S_{2}S_{3}$, $S_{1}I_{2}S_{3}$, $S_{1}S_{2}I_{3}$ with probabilities $f_{i}^{\left(1\right)}$, $f_{i}^{\left(2\right)}$, $f_{i}^{\left(3\right)}$ [36], which are:

(1) $$f_{i}^{\left(1\right)}\left(t\right)=1-\prod_{j=1}^{N}\left(1-\beta_{1}A_{ji}s_{j}^{1}\left(t\right)\right)$$

(2) $$\begin{array}{c} f_{i}^{\left(2\right)}\left(t\right)=1-\prod_{j=1}^{N}\left(1-\beta_{2}A_{ji}s_{j}^{2}\left(t\right)\right) \end{array}$$

(3) $$\begin{array}{c} f_{i}^{\left(3\right)}\left(t\right)=1-\prod_{j=1}^{N}\left(1-\beta_{3}A_{ji}s_{j}^{3}\left(t\right)\right) \end{array}$$

The variable *N* denotes the total number of nodes in the network. *A* is the adjacency matrix of the network. $A_{ji}=1$ indicates that individual *i* has an edge directed towards individual *j*, while $A_{ji}=0$ signifies that there is no directed edge between them. The variables $s_{j}^{1}\left(t\right)$, $s_{j}^{2}\left(t\right)$, and $s_{j}^{3}\left(t\right)$, respectively, indicate whether individual *j* is in an infected state with $Inf_{1}$, $Inf_{2}$, and $Inf_{3}$ at time *t*. If individual *j* is in an infected state, we have $s_{j}^{1}\left(t\right)=1$, otherwise $s_{j}^{1}\left(t\right)=0$. Similarly, $s_{j}^{2}\left(t\right)$ and $s_{j}^{3}\left(t\right)$ are defined.

The spreading mechanism operates as follows: Every node in states $I_{1}S_{2}S_{3}$ transmits information $inf_{1}$ to each of its adjacent nodes at time *t*. Each of these attempts has an independent success rate, represented by the probability $\beta_{1}$. Concurrently, nodes in states $S_{1}I_{2}S_{3}$ and $S_{1}S_{2}I_{3}$ also aim to disseminate information $Inf_{2}$ and $Inf_{3}$ to each of their neighboring nodes with probabilities $\beta_{2}$ and $\beta_{3}$, respectively. Therefore, $f_{i}^{\left(1\right)}$, $f_{i}^{\left(2\right)}$, and $f_{i}^{\left(3\right)}$ represent the probabilities that a given node *i* receives information $Inf_{1}$, $Inf_{2}$, and $Inf_{3}$ from its neighboring nodes.

**Figure 1:**
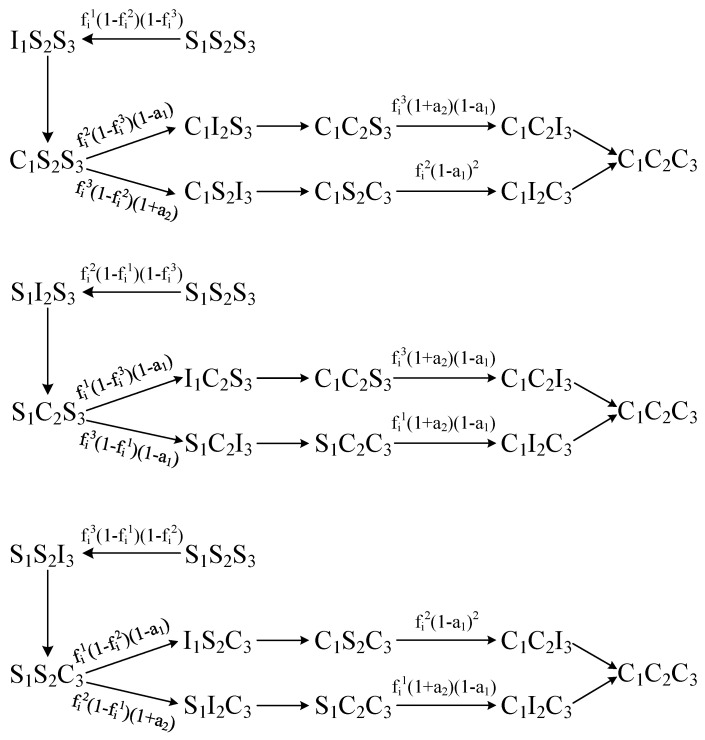
The state transition diagram for prioritizing the spreading of $Inf_{1}$, $Inf_{2}$ and $Inf_{3}$.

We construct a state transition diagram for the nodes, with Figure 1 represents the state transition diagram for prioritizing the reception of $Inf_{1}$, $Inf_{2}$, and $Inf_{3}$.

According to state transmission, we use the MMCA to describe the collaborative evolution process of all information sources. MMCA pivots on the fundamental principles of Markov chains, where the future state of a system is dependent solely on its current state. At the heart of this approach is the consideration of each individual in the population as a distinct entity, with a set of possible states. The transitions between these states are governed by probabilities:

(4) $$\begin{array}{c} \left\{\begin{array}{c} P_{i}^{I_{1}S_{2}S_{3}}\left(t+1\right)=P_{i}^{S_{1}S_{2}S_{3}}\left(t\right)f_{i}^{1}\left(1-f_{i}^{2}\right)\left(1-f_{i}^{3}\right)+P_{i}^{I_{1}S_{2}S_{3}}\left(t\right)\left(1-r_{i}^{1}\right)\left(1-f_{i}^{2}\right)\left(1-f_{i}^{3}\right)\\ P_{i}^{S_{1}I_{2}S_{3}}\left(t+1\right)=P_{i}^{S_{1}S_{2}S_{3}}\left(t\right)f_{i}^{2}\left(1-f_{i}^{1}\right)\left(1-f_{i}^{3}\right)+P_{i}^{S_{1}I_{2}S_{3}}\left(t\right)\left(1-r_{i}^{2}\right)\left(1-f_{i}^{1}\right)\left(1-f_{i}^{3}\right)\\ P_{i}^{S_{1}S_{2}I_{3}}\left(t+1\right)=P_{i}^{S_{1}S_{2}S_{3}}\left(t\right)f_{i}^{3}\left(1-f_{i}^{1}\right)\left(1-f_{i}^{2}\right)+P_{i}^{S_{1}S_{2}I_{3}}\left(t\right)\left(1-r_{i}^{3}\right)\left(1-f_{i}^{1}\right)\left(1-f_{i}^{2}\right)\\ P_{i}^{C_{1}S_{2}S_{3}}\left(t+1\right)=P_{i}^{I_{1}S_{2}S_{3}}\left(t\right)r_{i}^{1}\left(1-f_{i}^{2}\right)\left(1-f_{i}^{3}\right)+P_{i}^{C_{1}S_{2}S_{3}}\left(t\right)\left(1-f_{i}^{2}\right)\left(1-f_{i}^{3}\right)\\ P_{i}^{S_{1}C_{2}S_{3}}\left(t+1\right)=P_{i}^{S_{1}I_{2}S_{3}}\left(t\right)r_{i}^{2}\left(1-f_{i}^{1}\right)\left(1-f_{i}^{3}\right)+P_{i}^{S_{1}C_{2}S_{3}}\left(t\right)\left(1-f_{i}^{1}\right)\left(1-f_{i}^{3}\right)\\ P_{i}^{S_{1}S_{2}C_{3}}\left(t+1\right)=P_{i}^{S_{1}S_{2}I_{3}}\left(t\right)r_{i}^{3}\left(1-f_{i}^{1}\right)\left(1-f_{i}^{2}\right)+P_{i}^{S_{1}S_{2}C_{3}}\left(t\right)\left(1-f_{i}^{1}\right)\left(1-f_{i}^{2}\right)\\ P_{i}^{I_{1}I_{2}S_{3}}\left(t+1\right)=P_{i}^{S_{1}I_{2}S_{3}}\left(t\right)\left(1-r_{i}^{2}\right)f_{i}^{1}\left(1-f_{i}^{3}\right)\left(1-a_{1}\right)+P_{i}^{I_{1}S_{2}S_{3}}\left(t\right)\left(1-r_{i}^{1}\right)f_{i}^{2}\left(1-f_{i}^{3}\right)\left(1-a_{1}\right)\\ +P_{i}^{I_{1}I_{2}S_{3}}\left(t\right)\left(1-r_{i}^{1}\right)\left(1-r_{i}^{2}\right)\left(1-f_{i}^{3}\right)\\ P_{i}^{I_{1}S_{2}I_{3}}\left(t+1\right)=P_{i}^{I_{1}S_{2}I_{3}}\left(t\right)\left(1-r_{i}^{1}\right)\left(1-r_{i}^{3}\right)\left(1-f_{i}^{2}\right)+P_{i}^{S_{1}S_{2}I_{3}}\left(t\right)\left(1-r_{i}^{3}\right)f_{i}^{1}\left(1-f_{i}^{2}\right)\left(1+a_{2}\right)\\ +P_{i}^{I_{1}S_{2}S_{3}}\left(t\right)\left(1-r_{i}^{1}\right)f_{i}^{2}\left(1-f_{i}^{3}\right)\left(1+a_{2}\right)\\ P_{i}^{S_{1}I_{2}I_{3}}\left(t+1\right)=P_{i}^{S_{1}I_{2}I_{3}}\left(t\right)\left(1-f_{i}^{1}\right)\left(1-r_{i}^{2}\right)\left(1-r_{i}^{3}\right)+P_{i}^{S_{1}S_{2}I_{3}}\left(t\right)\left(1-r_{i}^{3}\right)f_{i}^{2}\left(1-f_{i}^{1}\right)\left(1-a_{1}\right)\\ +P_{i}^{S_{1}I_{2}S_{3}}\left(t\right)\left(1-r_{i}^{2}\right)f_{i}^{3}\left(1-f_{i}^{1}\right)\left(1-a_{1}\right)\\ P_{i}^{C_{1}I_{2}S_{3}}\left(t+1\right)=P_{i}^{I_{1}I_{2}S_{3}}\left(t\right)r_{i}^{1}\left(1-r_{i}^{2}\right)\left(1-f_{i}^{3}\right)+P_{i}^{C_{1}S_{2}S_{3}}\left(t\right)f_{i}^{2}\left(1-f_{i}^{3}\right)\left(1-a_{1}\right)\\ +P_{i}^{C_{1}I_{2}S_{3}}\left(t\right)\left(1-r_{i}^{2}\right)\left(1-f_{i}^{3}\right)\\ P_{i}^{I_{1}C_{2}S_{3}}\left(t+1\right)=P_{i}^{I_{1}I_{2}S_{3}}\left(t\right)\left(1-r_{i}^{1}\right)r_{i}^{2}\left(1-f_{i}^{3}\right)+P_{i}^{S_{1}C_{2}S_{3}}\left(t\right)f_{i}^{1}\left(1-f_{i}^{3}\right)\left(1-a_{1}\right)\\ +P_{i}^{I_{1}C_{2}S_{3}}\left(t\right)\left(1-r_{i}^{1}\right)\left(1-f_{i}^{3}\right)\\ P_{i}^{I_{1}S_{2}C_{3}}\left(t+1\right)=P_{i}^{I_{1}S_{2}I_{3}}\left(t\right)\left(1-r_{i}^{1}\right)\left(1-f_{i}^{2}\right)r_{i}^{3}+P_{i}^{S_{1}S_{2}C_{3}}\left(t\right)f_{i}^{1}\left(1-f_{i}^{2}\right)\\ +P_{i}^{I_{1}S_{2}C_{3}}\left(t\right)\left(1-r_{i}^{1}\right)\left(1-f_{i}^{2}\right)\\ P_{i}^{C_{1}S_{2}I_{3}}\left(t+1\right)=P_{i}^{I_{1}S_{2}I_{3}}\left(t\right)r_{i}^{1}\left(1-f_{i}^{2}\right)\left(1-r_{i}^{3}\right)+P_{i}^{C_{1}S_{2}S_{3}}\left(t\right)\left(1-f_{i}^{2}\right)f_{i}^{3}\left(1+a_{2}\right)\\ +P_{i}^{C_{1}S_{2}I_{3}}\left(t\right)\left(1-f_{i}^{2}\right)\left(1-r_{i}^{3}\right)\\ P_{i}^{S_{1}I_{2}C_{3}}\left(t+1\right)=P_{i}^{S_{1}I_{2}C_{3}}\left(t\right)\left(1-f_{i}^{1}\right)\left(1-r_{i}^{2}\right)+P_{i}^{S_{1}S_{2}C_{3}}\left(t\right)\left(1-f_{i}^{1}\right)f_{i}^{2}\left(1-a_{1}\right)\\ +P_{i}^{S_{1}I_{2}I_{3}}\left(t\right)\left(1-f_{i}^{1}\right)\left(1-r_{i}^{2}\right)r_{i}^{3}\\ P_{i}^{S_{1}C_{2}I_{3}}\left(t+1\right)=P_{i}^{S_{1}C_{2}I_{3}}\left(t\right)\left(1-f_{i}^{1}\right)\left(1-r_{i}^{3}\right)+P_{i}^{S_{1}I_{2}I_{3}}\left(t\right)\left(1-f_{i}^{1}\right)r_{i}^{2}\left(1-r_{i}^{3}\right)\\ +P_{i}^{S_{1}C_{2}S_{3}}\left(t\right)\left(1-f_{i}^{1}\right)f_{i}^{3}\left(1+a_{2}\right)\\ P_{i}^{C_{1}C_{2}S_{3}}\left(t+1\right)=P_{i}^{I_{1}C_{2}S_{3}}\left(t\right)r_{i}^{1}\left(1-f_{i}^{3}\right)+P_{i}^{C_{1}I_{2}S_{3}}\left(t\right)r_{i}^{2}\left(1-f_{i}^{3}\right)\\ +P_{i}^{C_{1}C_{2}S_{3}}\left(t\right)\left(1-f_{i}^{3}\right)\\ P_{i}^{S_{1}C_{2}C_{3}}\left(t+1\right)=P_{i}^{S_{1}C_{2}C_{3}}\left(t\right)\left(1-f_{i}^{1}\right)+P_{i}^{S_{1}I_{2}C_{3}}\left(t\right)\left(1-f_{i}^{1}\right)r_{i}^{2}\\ +P_{i}^{S_{1}C_{2}I_{3}}\left(t\right)\left(1-f_{i}^{1}\right)r_{i}^{3}\\ P_{i}^{C_{1}S_{2}C_{3}}\left(t+1\right)=P_{i}^{I_{1}S_{2}C_{3}}\left(t\right)r_{i}^{1}\left(1-f_{i}^{2}\right)+P_{i}^{C_{1}S_{2}C_{3}}\left(t\right)\left(1-f_{i}^{2}\right)\\ +P_{i}^{C_{1}S_{2}I_{3}}\left(t\right)\left(1-f_{i}^{2}\right)r_{i}^{3}\\ P_{i}^{I_{1}I_{2}I_{3}}\left(t+1\right)=P_{i}^{I_{1}I_{2}I_{3}}\left(t\right)\left(1-r_{i}^{1}\right)\left(1-r_{i}^{2}\right)\left(1-r_{i}^{3}\right)+P_{i}^{S_{1}I_{2}I_{3}}\left(t\right)f_{i}^{1}\left(1-r_{i}^{2}\right)\left(1-r_{i}^{3}\right)\left(1-a_{1}\right)\left(1+a_{2}\right)\\ +P_{i}^{I_{1}S_{2}I_{3}}\left(t\right)\left(1-r_{i}^{1}\right)f_{i}^{2}\left(1-r_{i}^{3}\right){\left(1-a_{1}\right)}^{2}+P_{i}^{I_{1}I_{2}S_{3}}\left(t\right)\left(1-r_{i}^{1}\right)\left(1-r_{i}^{2}\right)f_{i}^{3}\left(1-a_{1}\right)\left(1+a_{2}\right)\\ P_{i}^{I_{1}I_{2}C_{3}}\left(t+1\right)=P_{i}^{S_{1}I_{2}C_{3}}\left(t\right)f_{i}^{1}\left(1-r_{i}^{2}\right)\left(1-a_{1}\right)\left(1+a_{2}\right)+P_{i}^{I_{1}S_{2}C_{3}}\left(t\right)\left(1-r_{i}^{1}\right)f_{i}^{2}{\left(1-a_{1}\right)}^{2}\\ +P_{i}^{I_{1}I_{2}I_{3}}\left(t\right)\left(1-r_{i}^{1}\right)\left(1-r_{i}^{2}\right)r_{i}^{3}+P_{i}^{I_{1}I_{2}C_{3}}\left(t\right)\left(1-r_{i}^{1}\right)\left(1-r_{i}^{2}\right)\\ P_{i}^{C_{1}I_{2}I_{3}}\left(t+1\right)=P_{i}^{I_{1}I_{2}I_{3}}\left(t\right)r_{i}^{1}\left(1-r_{i}^{2}\right)\left(1-r_{i}^{3}\right)+P_{i}^{C_{1}S_{2}I_{3}}\left(t\right)f_{i}^{2}\left(1-r_{i}^{3}\right){\left(1-a_{1}\right)}^{2}\\ +P_{i}^{C_{1}I_{2}S_{3}}\left(t\right)\left(1-r_{i}^{2}\right)f_{i}^{3}\left(1-a_{1}\right)\left(1+a_{2}\right)+P_{i}^{C_{1}I_{2}I_{3}}\left(t\right)\left(1-r_{i}^{2}\right)\left(1-r_{i}^{3}\right)\\ P_{i}^{C_{1}C_{2}I_{3}}\left(t+1\right)=P_{i}^{I_{1}C_{2}I_{3}}\left(t\right)r_{i}^{1}\left(1-r_{i}^{3}\right)+P_{i}^{C_{1}I_{2}I_{3}}\left(t\right)r_{i}^{2}\left(1-r_{i}^{3}\right)\\ +P_{i}^{C_{1}C_{2}S_{3}}\left(t\right)f_{i}^{3}\left(1-a_{1}\right)\left(1+a_{2}\right)+P_{i}^{C_{1}C_{2}I_{3}}\left(t\right)\left(1-r_{i}^{3}\right)\\ P_{i}^{C_{1}I_{2}C_{3}}\left(t+1\right)=P_{i}^{I_{1}I_{2}C_{3}}\left(t\right)r_{i}^{1}\left(1-r_{i}^{2}\right)+P_{i}^{C_{1}S_{2}C_{3}}\left(t\right)f_{i}^{2}{\left(1-a_{1}\right)}^{2}\\ +P_{i}^{C_{1}I_{2}I_{3}}\left(t\right)\left(1-r_{i}^{2}\right)r_{i}^{3}+P_{i}^{C_{1}I_{2}C_{3}}\left(t\right)\left(1-r_{i}^{2}\right)\\ P_{i}^{I_{1}C_{2}C_{3}}\left(t+1\right)=P_{i}^{S_{1}C_{2}C_{3}}\left(t\right)f_{i}^{1}\left(1-a_{1}\right)\left(1+a_{2}\right)+P_{i}^{I_{1}I_{2}C_{3}}\left(t\right)\left(1-r_{i}^{1}\right)r_{i}^{2}\\ +P_{i}^{I_{1}C_{2}I_{3}}\left(t\right)\left(1-r_{i}^{1}\right)r_{i}^{3}+P_{i}^{I_{1}C_{2}C_{3}}\left(t\right)\left(1-r_{i}^{1}\right)\\ P_{i}^{C_{1}C_{2}C_{3}}\left(t+1\right)=P_{i}^{I_{1}C_{2}C_{3}}\left(t\right)r_{i}^{1}+P_{i}^{C_{1}I_{2}C_{3}}\left(t\right)r_{i}^{2}+P_{i}^{C_{1}C_{2}I_{3}}\left(t\right)r_{i}^{3}\\ P_{i}^{S_{1}S_{2}S_{3}}\left(t+1\right)=1-Above \end{array}\right. \end{array}$$

where $P_{i}^{I_{1}S_{2}S_{3}}\left(t+1\right)$ represents the probability of node *i* in the $I_{1}S_{2}S_{3}$ state at time t+1. A node is set in one of all different states, so $P_{i}^{S_{1}S_{2}S_{3}}\left(t+1\right)=1-Above$. The epidemic threshold is the critical point for information explosion. To determine the threshold, we first simplify Equation (4) to a general expression $\vec{P}\left(t+1\right)=g\left(\vec{P}\left(t\right)\right)$, where

(5) $$\begin{array}{cc} \vec{P}\left(t\right) & =({\vec{P}}^{I_{1}S_{2}S_{3}}\left(t\right),{\vec{P}}^{S_{1}I_{2}S_{3}}\left(t\right),{\vec{P}}^{S_{1}S_{2}I_{3}}\left(t\right),{\vec{P}}^{S_{1}I_{2}I_{3}}\left(t\right),{\vec{P}}^{I_{1}S_{2}I_{3}}\left(t\right),{\vec{P}}^{I_{1}I_{2}S_{3}}\left(t\right),{\vec{P}}^{I_{1}S_{2}C_{3}}\left(t\right)\\ \; & {\vec{P}}^{I_{1}C_{2}S_{3}}\left(t\right),{\vec{P}}^{S_{1}I_{2}C_{3}}\left(t\right),{\vec{P}}^{C_{1}I_{2}S_{3}}\left(t\right),{\vec{P}}^{C_{1}S_{2}I_{3}}\left(t\right),{\vec{P}}^{S_{1}C_{2}I_{3}}\left(t\right),{\vec{P}}^{I_{1}I_{2}C_{3}}\left(t\right),\\ \; & {\vec{P}}^{I_{1}C_{2}I_{3}}\left(t\right),{\vec{P}}^{C_{1}I_{2}I_{3}}\left(t\right),{\vec{P}}^{I_{1}C_{2}C_{3}}\left(t\right),{\vec{P}}^{C_{1}I_{2}C_{3}}\left(t\right),{\vec{P}}^{C_{1}C_{2}I_{3}}\left(t\right),{\vec{P}}^{I_{1}I_{2}I_{3}}\left(t\right)) \end{array}$$

${\vec{P}}^{I_{1}S_{2}S_{3}}\left(t\right)=\left(P_{1}^{I_{1}S_{2}S_{3}}\left(t\right),P_{2}^{I_{1}S_{2}S_{3}}\left(t\right),\ldots ,P_{N}^{I_{1}S_{2}S_{3}}\left(t\right)\right)$ and others are similarly.

**Theorem 1.**  *The system is asymptotically stable at $\vec{P}\left(t\right)=\vec{0}$ when the eigenvalues of $\nabla g(\vec{0})$ are less than 1 in absolute value, where Jacobian matrix is calculated as follows:*

(6) $${\left.J={\left[\nabla g\left(\vec{0}\right)\right]}_{m,n}=\frac{\partial g_{m,t+1}}{\partial P_{n,t}}\right|}_{{\vec{P}}_{n}=\vec{0}}.$$

In order to compute the epidemic threshold, it is necessary to solve the Jacobian matrix of the system. Based on $\vec{P}=\vec{0}$, the eigenvalues of its Jacobian matrix are as follows:

(7) $$\left\{\begin{array}{c} \left(1-\gamma_{1}\right)E+\beta_{1}A\\ \left(1-\gamma_{2}\right)E+\beta_{2}A\\ \left(1-\gamma_{3}\right)E+\beta_{3}A\\ \left(1-\gamma_{1}\right)\left(1-\gamma_{2}\right)E\\ \left(1-\gamma_{1}\right)\left(1-\gamma_{3}\right)E\\ \left(1-\gamma_{2}\right)\left(1-\gamma_{3}\right)E\\ \left(1-\gamma_{1}\right)E\\ \left(1-\gamma_{2}\right)E\\ \left(1-\gamma_{3}\right)E \end{array}\right.$$

According to Theorem 1, there are

(8) $$\begin{array}{cc} \; & Max((|\left(1-\gamma_{1}\right)+\beta_{1}\lambda |,|\left(1-\gamma_{2}\right)+\beta_{2}\lambda |,|\left(1-\gamma_{3}\right)+\beta_{3}\lambda |,|\left(1-\gamma_{1}\right)\left(1-\gamma_{2}\right)|,\\ & |\left(1-\gamma_{1}\right)\left(1-\gamma_{3}\right)\left|,\right|\left(1-\gamma_{2}\right)\left(1-\gamma_{3}\right)\left|,\right|\left(1-\gamma_{1}\right)\left|,\right|\left(1-\gamma_{2}\right)\left|,\right|\left(1-\gamma_{3}\right)\left|)<1\right) \end{array}$$

where λ represents the maximum eigenvalue of the adjacency matrix. When the system attains stability, no information can spread through the network.

$\gamma_{1}$, $\gamma_{2}$, $\gamma_{3},\beta_{1},\beta_{2},\beta_{3}$ all range from 0 to 1. In the context of undirected networks, the adjacency matrix possesses the properties of irreducibility, non-negativity, and symmetry. In accordance with the Perron–Frobenius Theorem, its largest eigenvalue is a positive real number, therefore $\left(1-\gamma_{1}\right)+\beta_{1}\lambda >0$, $\left(1-\gamma_{2}\right)+\beta_{2}\lambda >0$ and $\left(1-\gamma_{3}\right)+\beta_{3}\lambda >0$ and condition (8) can be rewritten as follows:

(9) $$\left\{\begin{array}{c} \left(1-\gamma_{1}\right)+\beta_{1}\lambda <1\\ \left(1-\gamma_{2}\right)+\beta_{2}\lambda <1\\ \left(1-\gamma_{3}\right)+\beta_{3}\lambda <1 \end{array}\Rightarrow \left\{\begin{array}{c} \beta_{1}/\gamma_{1}<1/\lambda \\ \beta_{2}/\gamma_{2}<1/\lambda \\ \beta_{3}/\gamma_{3}<1/\lambda \end{array}\right.\right.$$

When $\beta_{i}/\gamma_{i}<1/\lambda$, the spreading of information within the network has completed. It is evident from the derived results that the epidemic threshold remains independent of the interaction factors.

## 4. Results and Analysis

In this section, we verify the influence of the relationships between different pieces of information on multiple information propagation processes separately on real-world networks and synthetic networks.

### 4.1. Experimental Datasets

We conduct simulations of the proposed model based on both real and synthetic networks. The dataset for the real network is extracted from the Twitter dataset. The details are as follows:

1. **Twitter:** Twitter serves as a platform for real-time global event tracking and discussions on trending topics. Users can engage in open, real-time conversations and interact with other users. The dataset is collected from 5000 users and their social circles, where users and their social relationships are represented as nodes and connections, respectively. The statistical characteristics of the Twitter network are shown in Table 2.
2. **Synthetic network:** The synthetic network is generated using the Barabási–Albert (BA) network [37] and consists of 1000 nodes. The parameter *m* is set to 3, indicating the number of connected edges when a new node is added.

### 4.2. Multiple Information Analysis

In this section, we explore the influence of interaction factors on propagation. Given that the emphasis of this section is on the interaction between pieces of information, we primarily observe the impact of changes in the values of $\alpha_{1}$ and $\alpha_{2}$ on the spreading, where $a_{1}\in \left[0,1\right]$, $a_{2}\in \left[0,\infty \right)$. This ensures the generality of the experiments as we randomly select five initial nodes for information propagation experiments with each experiment repeated 100 times, and the results averaged.

**Figure 2:**
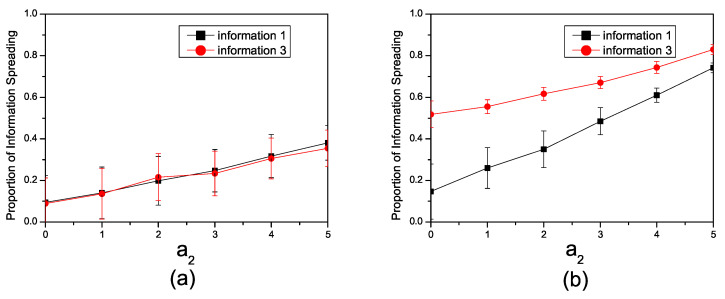
(**a**) The relationship in the synthetic network between the proportion of individuals spreading information and the enhancement factor $a_{2}$ with $\beta_{1}=0.1$ and $\beta_{3}=0.1$. (**b**) The relationship in the synthetic network between the proportion of individuals spreading information and enhancement factor $a_{2}$ with $\beta_{1}=0.1$, $\beta_{3}=0.3$.

In Figure 2a, we depict the relationship between the number of individuals involved in the spreading of information and the enhancement factor $a_{2}$ in the synthetic network, considering the parameters $\beta_{1}=0.1$, $\beta_{3}=0.1$. The information in the network mutually enhances each other. With an increase in $a_{2}$, the number of individuals involved in the spreading of information grows. The positive correlation between the enhancement factor and the number of individuals engaged in spreading information implies that as the strength of mutual enhancement increases, a larger segment of the network actively participates in the propagation of information.

Figure 2b illustrates the impact of facilitation factors on information propagation under different spreading probabilities. With $\beta_{1}=0.1$, $\beta_{3}=0.3$, we initially observed that the propagation range of both pieces of information increases with the strengthening of the facilitation factor. Additionally, the enhancement amplitude for $Inf_{1}$ is greater, while the enhancement amplitude for $Inf_{3}$ is smaller. In scenarios of mutual enhancement, the information with a higher inherent tendency to spread can act as a catalyst, facilitating the dissemination of less infectious information.

**Figure 3:**
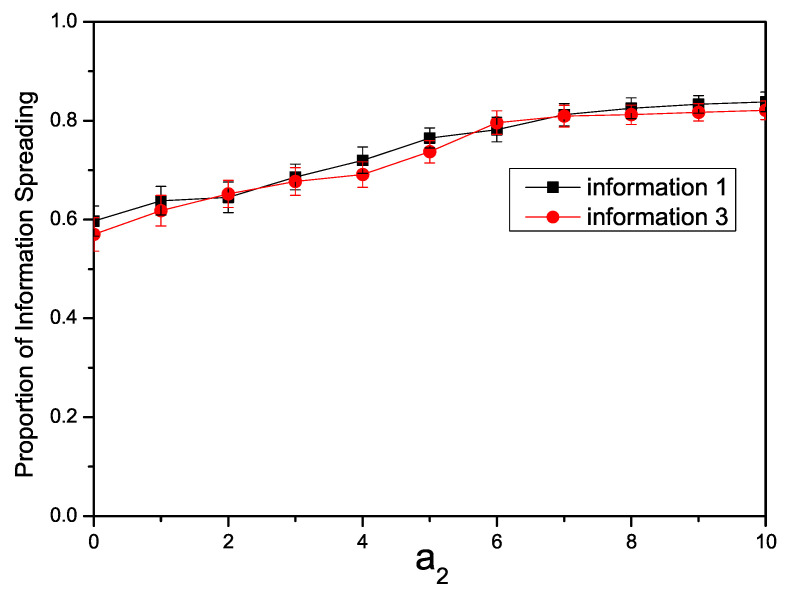
The relationship in the Twitter network between the proportion of individuals spreading information and the enhancement factor $a_{2}$ with $\beta_{1}=0.08$, $\beta_{3}=0.08$.

Figure 3 illustrates the variation in individuals participating in information propagation in the Twitter network under different enhancement factors. Given the structural disparities between the Twitter network and a synthetic network, we set the infection rate $\beta_{1}=0.08$, $\beta_{3}=0.08$. It can be observed that with an increase in the enhancement factor, the propagation range of $Inf_{1}$ and $Inf_{3}$ becomes larger. These findings align with the results observed in studies of synthetic networks.

**Figure 4:**
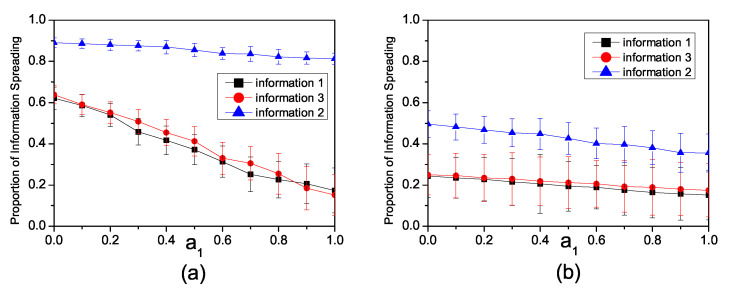
(**a**) The relationship in the synthetic network between the proportion of individuals spreading information and the inhibiting factor $a_{1}$ with $\beta_{1}=0.15$, $\beta_{3}=0.15$, $\beta_{2}=0.3$. (**b**) The relationship in the Twitter network between the proportion of individuals spreading information with the inhibiting factor $a_{1}$, $\beta_{1}=0.08$, $\beta_{3}=0.08$ and $\beta_{2}=0.3$.

To investigate the impact of the inhibition factor, we disregard the enhancement relationship between $Inf_{1}$ and $Inf_{3}$ by setting $a_{2}=0$. Figure 4a illustrates the influence of the inhibition factor on information propagation in a synthetic network. We set the infection rates as $\beta_{1}=0.15$, $\beta_{3}=0.15$, $\beta_{2}=0.3$. When the inhibition factor $a_{1}=0$, there is no competition among the three pieces of information. Therefore, all three pieces of information can be spread freely. As the inhibition factor increases, the propagation of all three pieces of information becomes constrained.

Figure 4b illustrates the impact of the inhibition factor $a_{1}$ on information propagation in the Twitter network. We set the infection rates as $\beta_{1}=0.08$, $\beta_{3}=0.08$ and $\beta_{2}=0.3$. When $a_{1}=0$, the influence range of all three pieces of information, which propagate freely, is significantly large. However, as $a_{1}$ increases, the propagation range of information with lower infection rates notably decreases, while the impact on information with higher infection rates is comparatively lower.

**Figure 5:**
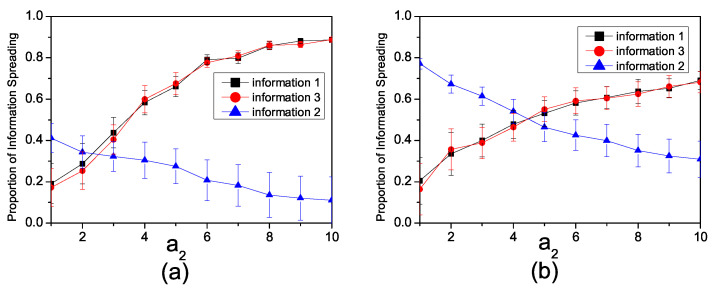
(**a**) The relationship in the synthetic network between the proportion of individuals spreading information and enhancement factor $a_{2}$ with $\beta_{1}=0.15$, $\beta_{3}=0.15$, $\beta_{2}=0.3$, $a_{1}=1$. (**b**) The relationship in the Twitter network between the proportion of individuals spreading information and the enhancement factor $a_{2}$ with $\beta_{1}=0.08$, $\beta_{3}=0.08$, $\beta_{2}=0.3$, $a_{1}=1$.

Figure 5a illustrates the variation in information propagation with changes in $a_{2}$ when both inhibition and enhancement factors coexist in a synthetic network. It can be observed that when the infection rates $\beta_{1}=0.15$, $\beta_{3}=0.15$ and $\beta_{2}=0.3$, and the inhibition factor is $a_{1}=1$, the propagation range of $Inf_{2}$ decreases with an increase in the enhancement factor. In contrast, the propagation ranges of $Inf_{1}$ and $Inf_{3}$ increase with the enhancement factor.

Figure 5b illustrates the variation in information propagation with changes in $a_{2}$ when both inhibition and enhancement factors coexist in the Twitter network. The infection rate is set as $\beta_{1}=0.08$, $\beta_{3}=0.08$ and $\beta_{2}=0.3$. When $a_{2}$ is small, the number of individuals spreading $Inf_{1}$ and $Inf_{3}$ is lower than that of $Inf_{2}$. However, as $a_{2}$ continues to increase, the number of individuals spreading $Inf_{1}$ and $Inf_{3}$ gradually surpasses the number of individuals spreading $Inf_{2}$. This dynamic signifies a critical turning point—when the enhancement factor is small, information with a higher infection rate impedes the dissemination of information with a lower infection rate. However, as the enhancement factor increases, a more complex interplay emerges. Information with a higher infection rate becomes subject to joint inhibition by multiple pieces of information characterized by lower influence probabilities.

**Figure 6:**
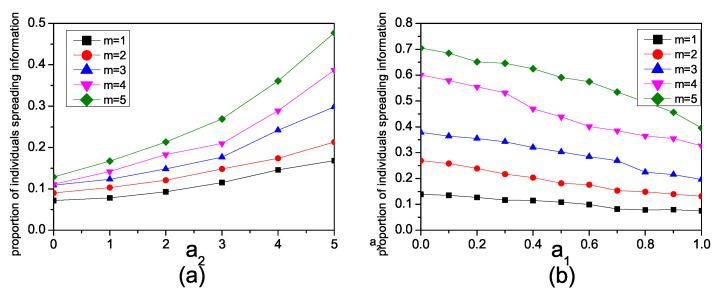
(**a**) The relationship in the synthetic network between the proportion of individuals spreading $Inf_{1}$ and the parameter *m* with $\beta_{1}=0.1$, $\beta_{3}=0.1$. (**b**) The relationship in the synthetic network between the proportion of individuals spreading $Inf_{2}$ and the inhibiting factor $a_{1}$ with $\beta_{1}=0.15$, $\beta_{3}=0.15$, $\beta_{2}=0.3$ and different *m*.

Next, we explore the impact of enhancement and inhibiting factors on information spreading in networks constructed with different BA model parameters *m*. Figure 6a illustrates the changes in the propagation of $Inf_{1}$ in synthetic networks for different *m* values. The infection rate is set as $\beta_{1}=0.1$, $\beta_{3}=0.1$. When *m* is small, the spreading of information faces significant challenges. Sparse networks typically feature limited connectivity among nodes, which hinders the efficient spread of information. In such scenarios, even with an increase in the enhancement factor, the impact on information propagation remains minimal. As *m* increases, the number of individuals involved in the propagation of $Inf_{1}$ gradually increases. The amplifying effect of the enhancement factor $a_{2}$ also becomes more pronounced, indicating that improving the connectivity of the network has a positive impact on information propagation.

Figure 6b illustrates the varying impact of the inhibition factor $a_{1}$ on the propagation of $Inf_{2}$ in synthetic networks for different values of *m*. The infection rate is set as $\beta_{1}=0.15$, $\beta_{3}=0.15$ and $\beta_{2}=0.3$. When *m* is large, an increase in the inhibition factor leads to a gradual reduction in the proportion of individuals involved in the diffusion of $Inf_{2}$. Conversely, when *m* is small, the influence of an increased inhibition factor on the diffusion of $Inf_{2}$ is small. These findings suggest that in networks with higher connectivity, the inhibition factor has a more significant impact on the propagation of $Inf_{2}$, while in networks with lower connectivity, the effect of the inhibition factor on $Inf_{2}$ propagation is less pronounced.

## 5. Conclusions

In this paper, we propose a SIC multi-information spreading model that incorporates enhancement and inhibiting factors to analyze their influence on the spread of multiple pieces of information. Through our experiments, several significant findings have emerged: (1) When multiple pieces of information mutually enhance each other, information with a higher infection rate can amplify the spread of information with a lower infection rate. This highlights the interplay between different pieces of information and how they can synergistically contribute to the overall spread. (2) The presence of competition among pieces of information hinders their spread. Moreover, the lower the infection rate of a piece of information, the more it is inhibited by competing information. This emphasizes the impact of competition on the spreading of individual pieces of information. (3) In cases where there is cooperation and competition among pieces of information, the one with a higher infection rate exerts a dominant influence on information spreading. This showcases the power dynamics at play when multiple pieces of information interact within a network. (4) In sparse networks with limited connectivity, the challenges in spreading information are more pronounced. Additionally, the impact of enhancement and inhibiting factors on information transmission becomes less significant in such networks. Overall, we provide valuable insights into understanding and predicting the dynamics of multi-information spreading in various contexts.

## Figures and Tables

**Table 1:** Symbols and definitions.

Symbols	Definitions
Inf	Information
$\beta_{i}$	Infection rate of $inf_{i}$
$\gamma_{i}$	Completed rate of $inf_{i}$
$a_{1}$	The influence parameter of the competitive information, and satisfies $a_{1}\in \left[0,1\right]$
$a_{2}$	The influence parameter of the cooperative information, and satisfies $a_{2}\in \left[0,\infty \right)$
V	The set of nodes
E	The set of edges
N	The number of nodes
M	The number of information
A	The adjacency matrix of the network
λ	The largest eigenvalue of matrix A
$S_{i}$	Susceptible state for information *i*
$I_{i}$	Infected state for information *i*
$C_{i}$	Completed state for information *i*

**Table 2:** Features of the Twitter network.

Number of Nodes	Number of Edges	Average Path Length	Clustering Coefficient
5000	185,433	3.2597	0.1178

## Data Availability

The data presented in this study are available on request from the corresponding author. The data are not publicly available due to privacy.
